# Van der Waals two-color infrared photodetector

**DOI:** 10.1038/s41377-021-00694-4

**Published:** 2022-01-02

**Authors:** Peisong Wu, Lei Ye, Lei Tong, Peng Wang, Yang Wang, Hailu Wang, Haonan Ge, Zhen Wang, Yue Gu, Kun Zhang, Yiye Yu, Meng Peng, Fang Wang, Min Huang, Peng Zhou, Weida Hu

**Affiliations:** 1 State Key Laboratory of Infrared Physics, Shanghai Institute of Technical Physics, Chinese Academy of Sciences, Shanghai, 200083 China; 2 University of Chinese Academy of Sciences, Beijing, 100049 China; 3 Hubei Yangtze Memory Labs, School of Optical and Electronic Information and Wuhan National Laboratory for Optoelectronics, Huazhong University of Science and Technology, Wuhan, Hubei 430205 China; 4 State Key Laboratory of ASIC and Systems, School of Microelectronics, Fudan University, Shanghai, 200433 China

**Keywords:** Optical materials and structures, Electronics, photonics and device physics

## Abstract

With the increasing demand for multispectral information acquisition, infrared multispectral imaging technology that is inexpensive and can be miniaturized and integrated into other devices has received extensive attention. However, the widespread usage of such photodetectors is still limited by the high cost of epitaxial semiconductors and complex cryogenic cooling systems. Here, we demonstrate a noncooled two-color infrared photodetector that can provide temporal-spatial coexisting spectral blackbody detection at both near-infrared and mid-infrared wavelengths. This photodetector consists of vertically stacked back-to-back diode structures. The two-color signals can be effectively separated to achieve ultralow crosstalk of ~0.05% by controlling the built-in electric field depending on the intermediate layer, which acts as an electron-collecting layer and hole-blocking barrier. The impressive performance of the two-color photodetector is verified by the specific detectivity (D*) of 6.4 × 10^9^ cm Hz^1/2^ W^−1^ at 3.5 μm and room temperature, as well as the promising NIR/MWIR two-color infrared imaging and absolute temperature detection.

## Introduction

Two-color infrared (IR) technology can identify targets in a complex environment by using the multispectral features of targets, and this technique has been widely used in information technology, life sciences, aerospace, and other fields^1–5^. As this technology has been developed, the main research direction has become the integration of two-color detection into single pixels without complex optical components^6^ while solving the core problem of separating and detecting dual spectral information independently^7^. Early techniques for obtaining the information from two bands involved a photodetector unit composed of multiple pixels with different response spectra in the plane, but poor spatial consistency led to aberrations in imaging. In 1980, a structure based on a back-to-back concept with three electrodes was designed^8^ to solve the problem of spatial coexcitation, but this strategy results in poor temporal coherence during sequential detection, which makes it very difficult to detect a moving target^9^. Since then, the simultaneous temporal-spatial mode of two-color IR photodetectors has been a popular research topic and has been successfully implemented in HgCdTe (MCT)^10^, quantum wells^11^, type II superlattices^12^ and so on. However, these conventional two-color IR photodetectors require complex cryogenic cooling systems to reduce dark current^13^, and their active-layer materials are more or less facing lattice mismatch at the interface in the process of multilayer heteroepitaxy, against the requirement of low-crosstalk and miniaturization applications^7^. Therefore, there is much interest in new IR materials and structures that fill gaps in the existing field.

Two-dimensional (2D) materials have shown infinite potential for use in future IR photodetectors^14–17^. Different from conventional thin-film materials, layered 2D materials exhibit van der Waals (vdWs) bonding^18^ and can therefore be stacked in any number of layers regardless of lattice mismatching^19^, the deposition temperature and dangling surface bonds^20^, which makes them promising for integration with mature 3D material systems^21–26^. Therefore, the band at the interface of the vdWs heterojunction can change abruptly with sharp band edges, which makes it possible to assemble devices with well-designed bands^27,28^. More importantly, layered 2D materials do not incur defects induced by internal stress^29^; therefore, the dark current induced by crystal surface defects and thermal ionization is reduced, indicating high potential for use in IR photodetection at room temperature^30–34^. Here, we designed a vertically stacked back-to-back 2D/3D hybrid photodetector with a black phosphorus (bP)/molybdenum sulfide (MoS_2_)/silicon (Si) vdWs heterostructure for use in temporal-spatial coexisting NIR/MWIR two-color blackbody sensitive photodetectors with ultralow crosstalk of ~0.05% at room temperature. Two built-in electric fields with opposite directions were successfully introduced by constructing a p-n-p^-^ junction with a back-to-back structure, achieving the separation and detection of photogenerated carriers in two bands. Finite-element simulation was also utilized to optimize the band structure to reduce optical and electrical crosstalk. We demonstrated absolute temperature detection and IR imaging in two colors, indicating the reliability and application prospects of vdWs heterojunctions based on silicon technology in two-color photodetection.

## Results

### Design of the van der Waals two-color infrared photodetector

**Fig. 1:**
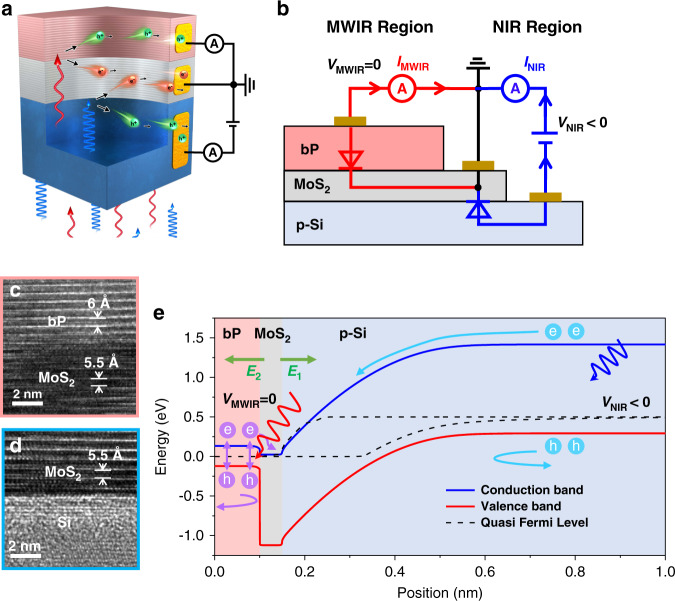
BP/MoS_2_/Si van der Waals two-color infrared detector concept. **a** Schematic of van der Waals two-color infrared photodetector, showing the working mode and external circuit of the photodetector. **b** Circuit configuration of device to demonstrate the performance of two-color photodetector. **c** Cross-sectional TEM image showing the bP/MoS_2_ interface. **d** Cross-sectional TEM image showing the Si/MoS_2_ interface. **e** Simulated energy band diagram with p-Si applied a negative voltage

A diagram of a two-color photodetector with a p-n-p structure is shown in Fig. 1a. P-type bP was combined with n-type MoS_2_ to form a bP/MoS_2_ p-n junction for MWIR detection; the bP/MoS_2_ p-n junction was transferred onto the etched window of p-type thin-film Si to form a MoS_2_/Si n-p junction for NIR detection, resulting in a vertical vdWs heterostructure of bP/MoS_2_/Si. The vdWs heterostructures were built by the dry transfer method^35^ with mechanical stripping and stacking. More details of the fabrication can be found in the Methods section and Supplementary Information. Operating under back irradiation conditions, the photodetector performance was measured by using the circuit configuration shown in Fig. 1b. In its vertical structure, n-type MoS_2_ plays the role of electron collection and hole barrier layer, while p-type Si and bP are used to absorb NIR and MWIR radiation, respectively, resulting in spatially consistent two-color detection. Under NIR illumination, photogenerated electron-hole pairs are separated by the built-in electric field (*E*_1_) of the MoS_2_/Si heterojunction, pushing electrons to flow through the electrode via MoS_2_ and holes to flow through the electrode via p-Si, while the same processes occur at the bP/MoS_2_ heterojunction under MWIR radiation. Figure 1e shows the simulated band diagram of bP/MoS_2_/Si with p-Si applied at a negative voltage, suggesting that its key advantage is generating and collecting photogenerated holes in the NIR and MWIR bands independently at the junctions of Si/MoS_2_ and bP/MoS_2_, respectively. This leads to little electrical crosstalk. A pseudocolor transmission electron micrograph (TEM) of a completed device is shown in Fig. 1c, d. The layered characteristics of bP and MoS_2_ are obvious, with the intervals of a single layer being 5.5 and 6 Å, respectively. The high-resolution TEM images show that the interface between the bp/MoS_2_ atomic layers was clean without any contamination or amorphous oxide being generated during the fabrication. Because bP is easily oxidized, we also measured oxygen at the bp/MoS_2_ interface, and EDS mapping results showed no oxygen or organic matter (Fig. S3a). The whole process involving the device was carried out in a nitrogen glove box, so almost no oxide layer or organic matter was present at the Si/MoS_2_ interface (Fig. S3b), which greatly improved the performance of the device.

### Two-color performance characterization

**Fig. 2:**
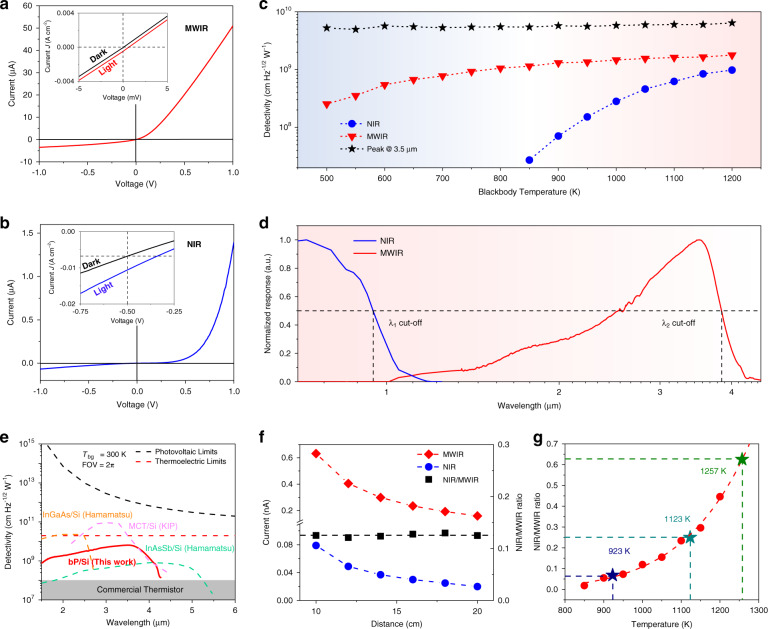
Two-color performance characterization and colorimetric temperature measurement. **a** Dark current characteristics of bP/MoS_2_ heterojunction photodiode in 300 K without illumination. Inset: the *J-V* characteristics of the device with or without a 1200 K blackbody source. **b** Dark current characteristics of Si/MoS_2_ heterojunction photodiode in 300 K without illumination. Inset: the *J-V* characteristics of the device with or without a 1200 K blackbody source. **c** Specific detectivity at different blackbody temperatures. **d** Normalized spectral response of the two-color photodetector during the back radiation. The 0.7–1.3 μm of the spectrum was measured by a grating spectrometer. The 1.3–4.5 μm of the spectrum was measured by FTIR. All FTIR and laser diode characterization were performed at a bias of 0 V (MWIR) and −0.5 V (NIR). **e** Specific detectivity as a function of wavelength measured for a bP/MoS_2_/Si heterojunction at room temperature as well as various commercially available and reported NIR/MWIR two-color photodetectors, including MCT/Si (Kunming Institute of Physics), InGaAs/Si (Hamamatsu K1713-08) and InAsSb/Si (Hamamatsu K1713-003). All FTIR and laser diode characterization was performed at a bias of 0 V. The black line in the figure is the performance limit of photovoltaic devices; the red line is the performance limit of thermoelectric device; the gray box is commercial thermistor. **f** Left axis, NIR and MWIR signals as a function of photodetector–blackbody distance. Right axis, the relationship between the NIR/MWIR ratio and photodetector–blackbody distance. **g** NIR/MWIR ratio depends on the blackbody temperature. Red dots show the measured NIR/MWIR ratio in each blackbody temperature

We separately evaluated the bP/MoS_2_ heterojunction of the photodetector with MWIR irradiation and the MoS_2_/Si heterojunction with NIR irradiation. The *I*–*V* curve characteristics of the bP/MoS_2_ heterojunction without illumination at 300 K are shown in Fig. 2a, illustrating a typical rectification characteristic of the p-n junction. The inset of Fig. 2a displays the relation between current density and voltage (*J-V* curves) under dark and 1200 K blackbody illumination, revealing a typical photovoltaic mode for the MWIR photoresponse. Figure 2b shows the *I*–*V* curve characteristics of the MoS_2_/Si heterojunction without illumination at 300 K. As a reverse bias voltage is applied, *E*_1_ is strengthened, and the depletion region is enlarged. The inset of Fig. 2b shows the *J-V* curves of the MoS_2_/Si heterojunction under dark conditions and 1200 K blackbody illumination at negative bias.

Specific detectivity D* is the key parameter for characterizing the photosensitive performance of IR photodetectors^36^ and can be calculated by the following equation:

1 $$D \ast = \frac{{\sqrt {A\Delta f} }}{{NEP}} = R\frac{{(A\Delta f)^{1/2}}}{{I_n}}$$

where *I*_*n*_ is the noise current, *R* is the responsivity, *A* is the effective area of the photodetector, and Δ*f* is the bandwidth. The noise current is dominated by several types of noise, including Johnson noise, shot noise and background fluctuation-induced noise. To date, no IR photodetector operating at room temperature can reach the performance limit of background fluctuation; therefore, the background fluctuation-induced noise can be neglected. However, the dominant source of noise current is different for our photodetector with two operating modes.

In MWIR band detection, the dominant noise in the photodetector is Johnson noise because there is no additional driving power. In this method, the calculation for photodiodes with zero bias usually requires the extraction of zero-bias resistance *R*_0_ (extracted from the *I*–*V* measurements)^36^:

2 $$D_0^ \ast = \frac{{\eta \lambda q}}{{hc}}\left( {\frac{{4kT}}{{R_0A}}} \right)^{{{{\mathrm{ - }}}}1/2}$$

where *η* is the external quantum efficiency, *λ* is the wavelength, *q* is the elementary charge, *h* is the Planck constant, *c* is the speed of light, *k* is the Boltzmann constant, *T* is the photodetector temperature, and $R_{{{\mathrm{0}}}}$ is the zero-bias resistance. From this, $D_0^ \ast$ of the MWIR photodetection at 1200 K blackbody is calculated to be 1.78 × 10^9^ cm Hz^1/2^ W^−1^.

For NIR, the noise in the photodetector under reverse bias is dominated by shot noise (much greater than Johnson noise)^36^. $D_R^ \ast$ can be calculated by:

3 $$D_{{{\mathrm{R}}}}^ \ast = \frac{{\eta \lambda q}}{{hc}}\left( {\frac{{A\Delta f}}{{2qI_D}}} \right)^{1/2}$$

where $I_D$ is the dark current. Therefore, the calculated $D_R^ \ast$ of the NIR photodetector with a 1200 K blackbody is 9.8 × 10^8^ cm Hz^1/2^ W^−1^. $D^ \ast$ of the device decreases with decreasing blackbody temperature, as shown in Fig. 2c. This is due to the redshift in the peak wavelength caused by the decrease in the blackbody temperature, and the radiation power is greatly reduced.

We further conducted spectral photoresponse measurements at room temperature for the two-color IR photodetector, as shown in Fig. 2d, with a Fourier transform IR (FTIR) spectrometer and grating spectrometer. The cut-off wavelengths in the NIR and MWIR ranges were 0.9 and 3.9 μm, respectively. The peak detectivity in the MWIR range was 6.4 × 10^9^ cm Hz^1/2^ W^−1^ at 3.5 μm (Fig. S6c) and was almost constant despite blackbody temperature changes, showing good stability and adaptability (Fig. 2c). The D^*^(λ) of the photodetector is comparable to that of commercialized noncooled NIR/MWIR two-color photodetectors, which indicates the application prospects of 2D vdWs heterostructures for use in IR photodetectors operating at room temperature (Fig. 2e). Figure S7 shows the measured noise current spectra of MWIR and NIR. According to the directly measured noise, the D* values calculated according to Eq. (1) were 3.51 × 10^9^ cm Hz^1/2^ W^−1^ (MWIR at 3.5 μm) and 3.34 × 10^8^ cm Hz^1/2^ W^−1^ (NIR), respectively. In this value, D^*^ calculated from *I*_*d*_ and *R*_*0*_ has good consistency. Moreover, the monochromatic photoresponse and low-temperature performance of the device was verified by the laser. The detailed information can be found in Fig. S9. Figure S8 shows the response time of the detector in microseconds and the linear dynamic range greater than 70 dB.

### Colorimetric temperature measurement

Two-color IR photodetectors are widely used for temperature measurement and provide more reliable and accurate detection than single-band photodetectors^2^. With increasing object temperature, the radiation intensity over the whole spectrum increases, and the peak shifts to shorter wavelengths, as shown by Planck’s law (Fig. S5a). According to Planck’s law under the Wien approximation, the monochromatic emission power M is:

4 $$M = \frac{{\varepsilon 2\pi hb\lambda ^{ - 5}\Delta \lambda }}{{\exp \left( {\frac{{hc}}{k}/\lambda T} \right)}}$$

where *ε* is the emissivity, *h* is the Planck constant, *c* is the light speed, *λ* is the wavelength, Δ*λ* is the wavelength width, *k* is the Boltzmann constant, and *T* is the photodetector temperature. For single temperature measurements, information on the emissivity and distance to the heat source is needed to determine the temperature of the object. However, in real measurements, the emissivity and distance to the heat source are often unknown. A two-color temperature measurement can eliminate the error associated with emittance and distance. Two-color temperature measurement is based on the ratio of two adjacent wavelengths of the IR radiation energy *R* to determine the temperature of the target object:

5 $$R = \frac{{M_1}}{{M_2}} = C_1exp\left[ {\left( {C_2T^{ - 1}} \right.} \right]$$

6 $$T = \frac{{C_2}}{{\ln R - \ln C_1}}$$

where *C*_1_ and *C*_2_ are the system constants. The reason for selecting adjacent wavelengths is that the epsilon of the adjacent wavelength of the emissivity rate is roughly the same; under certain conditions *ε*_1_/*ε*_2_, at the same time, and under the same detection range, its radiation power attenuation ratio is approximately the same. Therefore, the two-color photodetector can measure the absolute temperature by using the dual band information, regardless of the distance and emittance of the object, as shown in the schematic diagram of the temperature measurement setup in Fig. S4. Distance-dependent performance testing was performed to verify the influence of emission and radiation power attenuation at various distances on signal detection, and the result is shown in Fig. 2f. The NIR/MWIR signal ratio is almost a constant for a given temperature, independent of the radiation power and distance, so the value can correspond to the absolute temperature of the target at any distance. In addition, we varied the target temperature to verify the performance, as shown in Fig. 2g, revealing the corresponding relationship between the NIR/MWIR signal ratio and temperature. The signal ratio increased from 0.018 to 0.45 as the blackbody temperature increased from 850 to 1200 K. The temperatures of heat sources A and B were 923 and 1123 K, respectively, and the corresponding ratios obtained by the photodetector were 0.068 and 0.25. According to the fitting curve in Fig. 2g, corresponding ratios of 0.068 and 0.25 were calculated, and the detection temperatures were 930 and 1132 K, respectively, with the error being within the allowable range of the system. These results show that the two-color photodetector can remotely measure the temperature of a target heat source on the basis of the response ratio. Figure S5b shows the relationship between blackbody D^*^ and radiant power. Under the same blackbody temperature, the D^*^ of the device did not change with the change in blackbody radiation power. The operating frequency of the two-color photodetector reached 200 Hz, while the NIR/MWIR ratio remained constant, as shown in Fig. S12a. The repeatability of the measurement is shown in Fig. S13.

### Crosstalk simulation and characterization

For distinguishing signals, crosstalk is an important index of a two-color photodetector. The crosstalk in a two-color photodetector consists of optical crosstalk and electrical crosstalk^7^. Optical crosstalk refers to the response of the MWIR layer to NIR light that is not fully absorbed by the NIR absorption layer. Electric crosstalk refers to the nonequilibrium carrier diffusion between the two absorption layers. Specifically, there are two kinds of crosstalk in this device. *C*_*NIR to MWIR*_ (crosstalk of NIR to MWIR) is defined as the signal ratio of two junction signals of the NIR photodetector under MWIR radiation, which is mainly caused by electric crosstalk. *C*_*MWIR to NIR*_ (crosstalk of MWIR to NIR) is defined as the signal ratio of the MWIR photodetector under NIR radiation, which is mainly caused by combined optical and electric crosstalk (Fig. S14).

7 $$C_{NIR\,to\,MWIR} = {\int}_\lambda {C_{e\,NIR\,to\,MWIR}}$$

8 $$C_{MWIR\,to\,NIR} = {\int}_\lambda {C_{e\,MWIR\,to\,NIR}} + {\int}_\lambda {C_{o\,MWIR\,to\,NIR}}$$

**Fig. 3:**
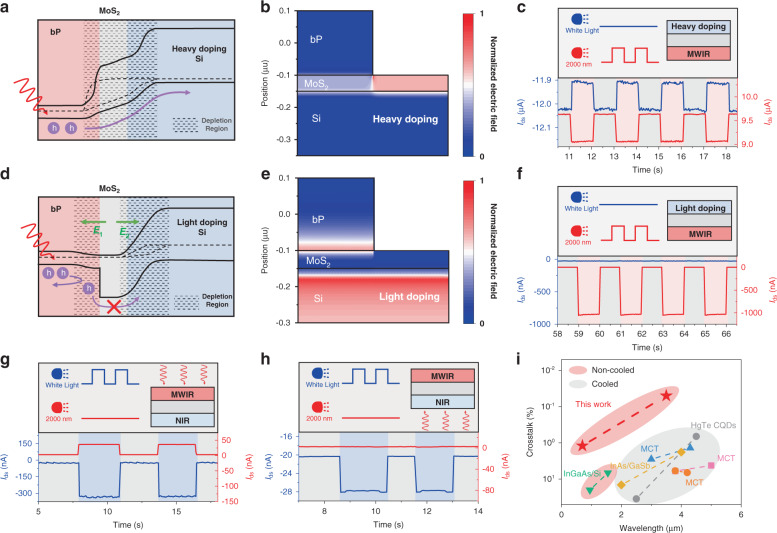
Ultralow crosstalk in bP/MoS_2_/Si two-color IR photodetector. **a** Band diagram and depletion region distribution of heavily doped Si two-color devices in working mode. **b** Electric field distribution of heavily doped Si two-color devices in working mode. **c** Photocurrent of heavily doped Si two-color photodetector under a modulated illumination source (λ = 2 μm). Red and blue line are MWIR and NIR photocurrents, respectively. **d** Band diagram and depletion region distribution of light doped Si two-color devices in working mode. **e** Electric field distribution of light doped Si two-color devices in working mode. **f** Photocurrent of light doped Si two-color photodetector under a modulated illumination source (λ = 2 μm). **g** Photocurrent of light doped Si two-color photodetector under a modulated illumination source (White light) in front radiation mode. **h** Photocurrent of light doped Si two-color photodetector under a modulated illumination source (White light) in back radiation mode. **i** Crosstalk performance of bP/MoS_2_/Si devices and various reported two-color photodetectors

For electric crosstalk, the electric field distribution and photocurrent distribution were studied by finite-element simulations. Different doping concentrations of Si can affect the internal electric field distribution. The band diagram for heavy Si doping is shown in Fig. 3a, and the *C*_*NIR to MWIR*_ for heavy Si doping under the same conditions was very large. Figure 3b shows that the heavily doped Si/MoS_2_ depletion region was mainly distributed in MoS_2_, leading to overlap of the two depletion regions, and the signal generated by the MWIR junction was received by both the MWIR and NIR electrodes. These test results show that the crosstalk could not be suppressed, as shown in Fig. 3c. For light Si doping (10^15^ cm^−3^), the band diagram is shown in Fig. 3d. The depletion regions of the NIR and MWIR photodiodes were distributed in Si and bP, respectively (Fig. 3e). The depletion region was effectively blocked by the intermediate layer, which formed a barrier to block photogenerated holes, resulting in ultralow electrical crosstalk in the device (Fig. 3f). Therefore, MWIR-to-NIR crosstalk of 1.2% and NIR-to-MWIR crosstalk of 0.05% were obtained from Eqs. (7) and (8), respectively.

The optical crosstalk was analysed by white light irradiation in front irradiation mode and back irradiation mode. In front irradiation mode, the MWIR device absorbed white light directly, so there was considerable optical crosstalk (Fig. 3g). The two-color photodetector in this paper used back irradiation, in which NIR absorber materials effectively absorbed NIR radiation and greatly reduced the optical crosstalk (Fig. 3h). Compared with the frontal incident mode, the *C*_*MWIR to NIR*_ decreased from 11.24% to 1.2% by integrating over the spectral coincidence area, as shown in Fig. S11a. To further verify the low crosstalk of the vdWs two-color photodetector, we compared the crosstalk in our device with that in other structures. Figure 3i shows the comparison of the two-color crosstalk in the vdWs two-color photodetector (red line) and commercial devices. The vdWs two-color photodetector exhibited ultralow two-color crosstalk compared to the integrated two-color photodetector based on conventional materials.

### Temporal-spatial coexisting two-color IR imaging

**Fig. 4:**
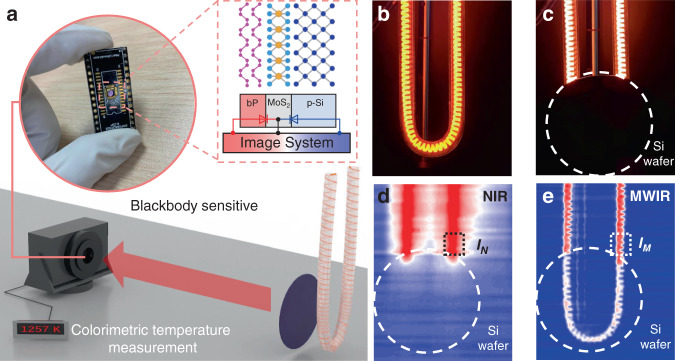
Temporal-spatial coexisting two-color IR imaging. **a** Schematic diagram of simultaneous mode two-color imaging system. Inset: optical image of two-color vdWs infrared photodetector. **b** Photograph of target (carbon fiber heating tube) was captured by a silica-based camera. **c** Photograph of target (carbon fiber heating tube covered by silicon wafer) was captured by a silica-based camera. **d** NIR images of objects behind a silicon wafer. The black dotted circle marks the position of the silicon wafer. The white dotted box indicates the location of photocurrent *I*_*N*_ extraction. **e** MWIR images of objects behind a silicon wafer. The white dotted circle marks the position of the silicon wafer. The white dotted box indicates the location of photocurrent *I*_*M*_ extraction

After calibrating the temperature recognition function of the device, a two-color imaging experiment was carried out. A blackbody-like radiant heat source was used as the target to verify the two-color detection capability of our device^37^. Figure 4a displays a schematic diagram of the imaging setup, and the two-color photodetector was used to replace the detecting chip of a camera. An image was acquired by two-dimensional scanning and converting the output voltage of each pixel into a gray value. Two output channels were used to ensure the simultaneous acquisition of both bands in the imaging. To better understand the two-color performance, we constructed a complex scene in which a Si wafer was placed in front of the heat target, as shown in Fig. 4b, c. Two opposite built-in electric fields can detect the photogenerated carriers from the two bands independently, which was enabled by the vertical structure. The photodetector showed high temporal and spatial consistency in the two-color detection. The NIR and MWIR imaging results were captured as shown in Fig. 4d, e. Here, the results are attributed to the high dynamic range imaging capability of the two-color photodetector, which is a key feature of high-quality imaging. On the other hand, the lower half of the target was not detected because the light does not penetrate Si, as shown in the NIR imaging results (Fig. 4d). Therefore, in a complex environment, a two-color photodetector can obtain more target information than single-color imaging and detect the temperature of the target at the same time as two-color imaging. The photocurrents *I*_*N*_ and *I*_*M*_ were extracted from the two-color image, and the signal ratio of the heating tube was 0.625. Based on Fig. 3c, the temperature of the heating tube was ~1257 K, which was very close to the actual temperature of 1300 K. Interestingly, commercial handheld IR thermometers could not identify the internal temperature of the resistance wire because the glass shell of the heating tube blocked the heat emitted by the carbon fiber heating wire (Fig. S17). These results show that a two-color image can provide more information than a single-band image.

## Discussion

In this study, we systematically investigated vdWs heterostructure-based multispectral detection to design and fabricate a two-color IR photodetector based on the p-n-p^-^ vertical structure of bP/MoS_2_/Si and found impressive room-temperature specific detectivity D^*^ of 6.4 × 10^9^ cm Hz^1/2^ W^−1^ at 3.5 μm under blackbody radiation. The photogenerated carriers in two bands are successfully separated by two opposite built-in electric fields. The ultralow crosstalk of 1.2% for MWIR-to-NIR and 0.05% for NIR-to-MWIR due to the filtering effect of thin-film Si and the barrier effect of MoS_2_ ensures the independent readout of the dual band information. Moreover, calibration of the response under both the NIR and MWIR bands can be used to determine the absolute temperature of a heat source regardless of the detection distance and emissivity. Simultaneous imaging of the NIR and MWIR bands and imaging temperature detection were demonstrated. Compatible with existing silicon technology, vdWs heterojunctions can enable room-temperature IR two-color detection because of their novel device structure and energy band design, which is expected to improve current multispectral IR detection technologies.

## Methods

### Device fabrication

The bP/MoS_2_/Si vdWs heterostructure was fabricated by the dry transfer method. The 2D materials were mechanically exfoliated from bulk materials supplied by Smart Elements, HQ graphene. A detailed description of this process is provided in Supplementary Section 1. The exfoliation and transfer processes were carried out in a N_2_-protected glovebox to decrease the oxidation of the material. Electrode contact was defined using electron beam lithography, and Cr/Au (15/80 nm) was deposited using thermal evaporation. Before measurement, a thin PMMA layer was coated to protect the device from exposure to air and water.

### Photodetector performance

The simulation structure consisted of a 200 nm p-type black phosphorus layer, a 50 nm n-type MoS_2_ layer, and a 200 nm p-type Si layer. The doping concentrations were assumed to be 5 × 10^15^ cm^−3^ black phosphorus, 1 × 10^17^ cm^−3^ MoS_2_, 1 × 10^18^ cm^−3^ Si (high doping), and 1 × 10^15^ cm^−3^ Si (low doping). The electronic characteristics were measured by an Agilent B2902A source. Blackbody testing was performed using a calibrated commercial blackbody furnace (HFY-206A). After the frequency was modulated by an optical chopper wheel, the photocurrent signal was converted into a voltage signal using a current preamplifier (Stanford Research Systems SR570) and recorded by a lock-in amplifier (Ametek Model 7270 DSP). The laser spectral response test was carried out by a combination of a continuous laser light source (YSL sc-pro) and gratings. The IR spectrum test adopted in this experiment was carried out by an FTIR spectrometer (Nicolet 8700), and globar light (1000 K blackbody) was used as the internal light source at room temperature. In all the photocurrent measurements, the laser (2000 nm) was focused on the device through a fixed optical path using a 20× objective lens (NA = 0.45) with a laser spot diameter of ~20 μm. The mid-IR laser responses of the photodetectors were tested by a mid-IR plasma laser with tuneable wavelengths (2.6, 3.1, 4, and 4.2 μm). The transient photocurrent response (at 2000 nm) was recorded using an oscilloscope (Tektronix DPO 5204) to analyse the photoresponse time. All measurements were performed at room temperature in an ambient environment. Electrical simulations were performed using the finite-element method.

## Supplementary information

supporting information
