# Supplementary material for: Van der Waals two-color infrared photodetector
Source: Light Sci Appl. 2022 Jan 2;11:6. doi: 10.1038/s41377-021-00694-4 (PMC8720310; doi:10.1038/s41377-021-00694-4)
Supplement: Supplementary file 1 — supporting information [file 41377_2021_694_MOESM1_ESM.docx]

**Supplementary Information:**

**Van der Waals Two-color Infrared Photodetectors**

Peisong Wu^1,2†^, Lei Ye^3†^, Lei Tong^3†^, Peng Wang^1*^, Yang Wang^1,4^, Hailu Wang^1,2^, Haonan Ge^1,2^, Zhen Wang^1,2^, Yue Gu^1,2^, Kun Zhang^1^, Yiye Yu^1,3^, Meng Peng^1,3^, Fang Wang^1^, Min Huang^1^, Peng Zhou^4*^, and Weida Hu^1,2*^

^1^State Key Laboratory of Infrared Physics, Shanghai Institute of Technical Physics, Chinese Academy of Sciences, Shanghai 200083, China

^2^University of Chinese Academy of Sciences, Beijing 100049, China

^3^School of Optical and Electronic Information and Wuhan National Laboratory for Optoelectronics, Huazhong University of Science and Technology, Wuhan, Hubei 430074, China

^4^State Key Laboratory of ASIC and System, School of Microelectronics, Fudan University, Shanghai 200433, China

^†^These authors contributed equally to this work.

*email: w_peng@mail.sitp.ac.cn, pengzhou@fudan.edu.cn, wdhu@mail.sitp.ac.cn

**Contents**

1. Fabrication of two-color photodetectors
2. Cross-sectional transmission electron micrograph (TEM) image of two-color photodetector and EDS mapping
3. Blackbody photoresponse measurement
4. Spectral response measurement
5. Noise test of device and system
6. Photodiode laser photoreponse performance
7. Two-color performance of bP/MoS_2_/Si vdWs heterojunction
8. Crosstalk changes at different work bias in two-color photodetectors
9. Heat source imaging and temperature measurement
10. **Two-color photodetectors fabrication.**

Fabrication of window in SiO_2_/p-Si wafer for back-to-back vdW heterostructure (p-Si resistivity 1-20 Ohm∙cm). First, PMMA is spun coating (3000 rpm 60 seconds) on SiO_2_/ P-Si wafer, and then heated on the hot plate at 150 ℃ for 10 min. Next, the Si corrosion window on the PMMA layer is used EBL to define, and then treated with a developer solution. Finally, the Si window on SiO_2_/Si is fabricated, and the prepared substrate is quickly transferred into the glove box to prevent Si oxidation. The process is shown in **Fig. S1a**. The PPC (poly propylene carbonate) is stripped from the Si substrate, placed on the PDMS, and then the PDMS is affixed upside down on the microscope slide. At the same time, mechanical stripping of bP and MoS_2_ onto SiO_2_/Si. Operating in a nitrogen glove box using a low-dimensional material transfer system, the PPC film was in contact with bP and heated to 40 ℃. After cooling to 30 ℃, the slide is lifted so that the bP sticks to the PPC film. This process is repeated to pick up MoS_2_ and transfer the bP /MoS_2_ heterojunction to the Si window. Under 120 ℃, the glass slide and PDMS can be removed after the PPC is melted on the surface of the heterojunction. Finally, PPC is removed in acetone and left from the wafer to form a bP /MoS_2_/Si heterojunction. The process is shown in **Fig. S1b**. Electrode contact is defined using electron beam lithography and Cr/Au is deposited by thermal evaporation. Optical micrograph of the device is show in **Fig. S2**.

**Fig. S1.** **Schematic diagram of corrosion process.** **a,** Si window wet etching process. **b,** Heterojunction transfer process.

**Fig. S2. Optical micrograph of the device.** **a,** Optical micrograph of the device. **b,** Detail of optical micrograph of the device. The red dotted line is black phosphorus. The yellow dotted area is molybdenum disulfide. The blue dotted area is the silicon window. **c,** Thickness of the bP, MoS_2_ and SiO_2_ measured by AFM (150, 50 and 140 nm, respectively).

**2.** **Cross-sectional transmission electron micrograph (TEM) image of bP/MoS_2_/Si vdW heterostructure and EDS mapping.**

The cross-sectional TEM and EDS mapping of the bP/MoS_2_/Si vdW heterostructure is shown in **Fig. S3a**. The results show that the material elements are evenly distributed and the lattice quality is good. The distribution of oxygen elements at the bP /MoS_2_ interface is characterized, suggesting that there is no oxygen element and carbon element at the interface. This indicates that the quality of the device is good. The distribution of oxygen element at Si/MoS_2_ interface is characterized (**Fig. S3b**), suggesting that there is no oxygen element and carbon element at the interface. The smooth Si interface indicates that a smooth interface can be formed by using BOE etching solution.

**Fig. S3.** **EDS mapping images of the interfaces** **a,** EDS mapping images of the MoS_2_/bP interfaces of the device. **b,** EDS mapping images of the MoS_2_/Si interfaces of the device.

**3. Blackbody photoresponse measurement.**

The photoresponse of infrared photodetector describes its photoelectric conversion ability, as one of the most important indicators of photoelectric devices. For photovoltaic detectors, the current photoresponse is usually expressed as the output short-circuit current signal generated by the device per unit of radiant power. The photoresponse of the photodetector is not only related to the incident light power, but also to the wavelength of the radiation source. So, it is necessary to calibrate the photoresponse of the device by using the radiation source with certain radiated power and wavelength distribution. Blackbody measurements are performed in the atmosphere at room temperature. The device is located at a distance from the aperture, which can vary with the slide. The total incident power (*P*) on the device surface can be calculated by formula:

$$P=\frac{\alpha\varepsilon\sigma(T^{4}-T_{0}^{4})A}{\pi L^{4}}A_{n}$$

Where *α* is the modulation factor, *ε* is the average emissivity of the blackbody radiation source (here, *ε* is 0.9 for our blackbody source), *σ* is the Stefan-Boltzmann constant. *T* is the temperature of the blackbody radiation source, *T_0_* is the room temperature (300 K). *L* is the distance between the aperture and device, *A* is the area of the blackbody radiation source, *A_n_* is the device area.

The photodetector absorbs the incident photons to generate the voltage signal, which is converted by the current preamplifier to obtain the final current signal *I*. Then, the current response rate *R* of the device can be calculated by *I*/*P*. The specific process of blackbody response test is shown in **Fig. S4**. In order to obtain more accurate current signals, a chopper will be introduced into the test optical path diagram to modulate the radiation spectrum, and the phase-locked amplifier will read the signal to filter system noise and background noise.

**Fig. S4. Diagram of two-color temperature measuring system.** The blackbody light source is irradiated on the device after mechanical frequency modulation. The signal is plugged into two preamplifiers respectively and then collected by the phase-locked amplifier.

**4. Spectral response test.**

Blackbody response test results in the overall response of the photodetector to a wide spectrum radiation source. In order to obtain the relationship between the photoresponse of the photodetector and the radiation wavelength, the spectral response test is required. The spectral response test can be obtained by using a monochromatic radiation source, and the response spectrum of the photodetector can be obtained by changing the wavelength of the radiation source. In this work, there are two kinds of spectral tests. The NIR part is measured by grating spectrometer, and the mid-infrared part is measured with a Fourier spectrometer. Grating spectrometer uses compound light to disperse through prism or diffraction grating to separate monochromatic light. The interferometric Spectrometer is generally realized based on Michelson interferometer and Fourier Transform signal processing system. For the experimental spectrum tests, they are conducted by using Nicolet 8700 spectrometer system. The composite infrared radiation light is emitted from the globar and other mid-infrared light sources and then enters the Michelson interferometer, in which the moving mirror of the interferometer moves at a certain rate, thus giving the interference light time-domain characteristics, and then irradiates to the sample surface to generate photocurrent signals. Finally, the required spectral response information is obtained after the inverse Fourier transform is done according to the time-domain characteristics of the sample signal.

With decreasing the blackbody source temperature, the radiation intensity in the whole spectrum is decreased and the peak is shifted to longer wavelengths, as Planck law (**Fig. S5a**). The proportion of the radiated power of different wavelengths in the total power of the blackbody light source at different temperatures is also different. At the same time, we change the distance between the device and the blackbody source, and carry out variable power test. The test results show that the *D^*^ (Blackbody)* of our two-color device is not related to the blackbody radiated power (**Fig. S5b)**.

**Fig. S5. a,** Blackbody radiation spectra at different temperatures calculated according to Planck's law. **b,** Specific detectivity are calculated under blackbody radiation, which is unrelated to power intensity.

Blackbody response test results in the overall response of the photodetector to a wide spectrum radiation source. In order to obtain the relationship between the responsivity of the detector and the radiation wavelength, spectral response test is required. In this paper, the grating spectrometer and FTIR are combined to test. In general, the blackbody response test obtains the blackbody responsivity *R_b_* and the blackbody detectivity *D^*^* (blackbody), and the FTIR test obtains the relative responsivity *R*^'^ (*λ*). So by calculating the factor *g*, we can get the peak responsivity *R* (*λ_p_*) and the peak detectivity *D*^*^ (*λ_p_*). Now let's show how we can calculate *g*.

$R^{`}(\lambda)=\frac{R(\lambda)}{R(\lambda_{p})}$ (2)

Because blackbody radiation has a continuous spectrum and the emissivity of each wavelength is different, the signal produced by the photodetector is the sum of the signals produced by each wavelength of radiation:

 (3)

g factor can pass the test of the relative response spectrum 𝑅` (𝜆) and blackbody radiation power distribution 𝜙 (𝜆) is calculated:

 (4)

The response spectrum obtained by Fourier spectrometer is only a relative response spectrum, and the response rate of current spectrum of the photodetector can be obtained after blackbody response calibration. The relationship between the peak current photoresponse *R*_λp_ and the blackbody photoresponse *R_b_* of the photodetector is as follows (**Fig. S6a**):

$R(\lambda_{p})=\frac{R_{b}}{g}$ (5)

g factor can pass the test of the relative response spectrum 𝑅 (𝜆) and blackbody radiation power distribution 𝜙 (𝜆) is calculated:

 (6)

Thus, the quantum efficiency spectrum of the photodetector can be further calculated as follows (**Fig. S6b**):

$\eta(\lambda)=\frac{R_{i}(\lambda)*hc}{q\lambda}$ (7)

The detectivity of our two-color photodetector starts approaching the performance of commercial epitaxial materials-based two-color photodetectors. A comparison between detectivity is show in **Fig. 2e**.

**Fig. S6.** **Spectral measurement of MWIR photodiode.** **a,** The spectral photoresponse of the MWIR photodiode. **b,** The spectral external quantum efficiency of the MWIR photodiode. **c,** The spectral specific detectivity of the MWIR photodiode at 1200 K blackbody.

**5. Device and system noise test.**

The system noise test results are shown in **Figure S7c**. The shape it exhibits is common in systems dominated by the generation–recombination noise. The noise measured by MWIR is 9×10^-26^ A^2^ Hz^-1/2^ (**Fig. S7a**). This is similar to previously reported photodiodes made of black phosphorus. The noise measured by NIR is 5×10^-26^ A^2^ Hz^-1/2^ (**Fig. S7b**). In order to verify the *D^*^* value of the two-color photodetector, we measured the noise equivalent power (NEP) of the device under flood illumination with a blackbody source.

$D^{*}=\frac{\sqrt{A\Delta f}}{NEP}=\frac{R\sqrt{A\Delta f}}{i_{n}}$ (7)

According to the directly measured noise current, the two-color *D^*^* is calculated as 3.51×10^9^ cm·Hz^1/2^·W^-1^ (MWIR) & 3.34×10^8^ cm·Hz^1/2^·W^-1^ (NIR). In this value, *D^*^* calculated by *I_d_* and *R_0_* has a good consistency.

**Fig. S7. a,** Spectral noise density of a bP/MoS_2_ heterojunction photodiode at a bias of 0 V. **b,** Spectral noise density of a Si/MoS_2_ heterojunction photodiode at a bias of -0.5 V. **c,** Spectral noise density of system noise.

**6. Photodiode laser photoreponse performance.**

We test the optoelectronic performance of the MWIR device, and the response speed of the MWIR device for 2 μm laser was about 50 μs (**Fig. S8a**). We test the optoelectronic performance, and the response speed for NIR region (Using 830 nm laser to measure) is about 20 μs (**Fig. S8c**).

**Fig. S8. Frequency response test and variable power response test of the device.** **a,** 90–10% rise and fall times measured with a λ = 2 μm illumination source of MWIR photodiode. **b,** Measured photocurrent as a function of incident illumination intensity at 2 μm of MWIR photodiode, and the corresponding LDR is 75.3 dB. **c,** 90–10% rise and fall times measured with a λ = 830 nm illumination source of NIR photodiode. **d,** Measured photocurrent as a function of incident illumination intensity at 830 nm of NIR photodiode, and the corresponding LDR is 104.7 dB.

Most photodetector applications, such as image sensors and photometers, require the extraction of detecting light intensity from photocurrent over a wide range of light intensity, so having a constant responsivity over a wide range of light intensity is an important feature of photodetectors. Linear dynamic range (LDR) is a performance value used to characterize the light intensity range of a photodetector with a constant response. It is always expressed as:

$\text{LDR}=20\log\frac{P_{\max}}{P_{low}}$ (8)

Where, *P*_max_ is the maximum value of incident light intensity in the linear region, and *P*_low_ is the minimum value of incident light intensity in the linear region. We conducted the LDR measurement via change the incident laser power. The linear range of MWIR photoresponse is from 2.7×10^-3^ mW mm^-2^ to 15.7 mW mm^-2^, and the corresponding LDR is 75.3 dB (**Fig. S8b**). The NIR responds linearly from 1.6×10^-5^ mW mm^-2^ to 2.76 mW mm^-2^, and the corresponding LDR is 104.7 dB (**Fig. S8d**).

In this work, a mid-infrared pulse laser is used to characterize the device's mid-infrared performance (**Fig. S9**). We use a tunable mid-infrared pulse laser as the light source. The photoresponse of 2.6 μm, 3.1 μm, 4 μm and 4.2 μm were tested respectively. The device is tested in a normal atmospheric environment. The device shows obvious photoresponse, and the calculated photoresponse is basically the same as that measured by FTIR spectrum. This demonstrates the reliability of device performance.

**Fig. S9. MWIR & NIR photoresponse characterization. a,** Photocurrent measured from a MWIR photodiode under a modulated illumination source (*λ* = 2.6 μm). **b,** Photocurrent measured from a MWIR photodiode under a modulated illumination source (*λ* = 3.1 μm). **c,** Photocurrent measured from a MWIR photodiode under a modulated illumination source (*λ* = 4 μm). **d,** Photocurrent measured from a MWIR photodiode under a modulated illumination source (*λ* = 4.2 μm). **e,** The photoresponse of the device at MWIR. f, Specific detectivity of the two-color photodetector under the monochromatic laser at the temperature of 200-300 K.

We tested and analyzed the performance of the device at lower temperatures, and the result was that the photoresponse of the device remained basically unchanged as the temperature decreased, while the dark current of the device decreased significantly. The temperature range we chose was between 200 K and 300 K, which can be achieved by the TEC stage. The test results show that the detector has better performance in low temperature environment. The *D^*^* of the detector at 300K are 1.11×10^9^ cm·Hz^1/2^·W^-1^ (NIR @ 830 nm) and 2.21×10^9^ cm·Hz^1/2^·W^-1^ (MWIR @ 2000 nm) respectively. As the temperature decreases, the dark current of the device decreases. The *D^*^* of the detector at 200K are 1.31×10^10^ cm·Hz^1/2^·W^-1^ (NIR @ 830 nm) and 2.05×10^10^ cm·Hz^1/2^·W^-1^ (MWIR @ 2000 nm) respectively. (**Fig. S9f)**

**7.** **Two-color performance of bP/MoS_2_/Si vdWs heterojunction.**

We design the back radiation for testing the device. Back radiation is used to meet standard operating conditions for conventional two-color photodetectors, while mode two-color photodetectors usually require two sets of readout circuits (**Fig. S10)**. Therefore, back radiation is used to maximize the light absorption of the two layers. The readout circuit diagram of the device is from J12 Series InAs Photodetectors of Judson Technologies.

**Fig. S10. Readout circuit of the two-color photodetector.**

We test the spectral response during the front radiation. The incident light first illuminates the MWIR layer and then the NIR layer. This causes the MWIR layer to respond to a large amount of NIR radiation, resulting in a large amount of optical crosstalk (**Fig. S11a**). The NIR absorbing material Si is located above BP and contacts the light first. So the spectral response from 0.7 μm to 1 μm is almost completely absorbed by Si, resulting in the bP response spectrum cut off exactly at about 1 um (**Fig. S11b**). In this regard, we also carried out a comparison test, placing bP above Si and contacting the light first (**Fig. S11c**). The test showed that bP could respond normally to the spectral response of 0.7 μm to 1 μm at this stage, results showed that the response decreased.

**Fig. S11. a,** Spectral response of the two-color photodetector during the front radiation. **b,** Schematic diagram of the two-color photodetector in the back radiation mode. **c,** Schematic diagram of the two-color photodetector in the front radiation mode.

We test the frequency response of the two-color device. The test frequency is modulated by a mechanical chopper. The results show that the dual-band photodetector can measure the temperature of the target heat source remotely by the response ratio. The operating frequency of the dual-band photodetector is up to 200Hz, and the NWIR/MWIR ratio remains unchanged, as shown in **Fig. S12a**.

**Fig. S12.** **a,** NIR/MWIR ratio depends on the operation frequency under different blackbody temperatures.

To verify the repeatability of the device. We fabricate three two-color photodetectors with the same structure, namely device 1, device 2 and device 3. The two-color performance of device 1 has been demonstrated in the text. The electrical and dichromatic properties of device 2 are shown in **Fig. S12**. Two-color temperature measurement experiment for the device 2 is also carried out, and the results are shown in **Fig. S13**.

**Fig. S13. Two-color IR thermometer. a,** Left axis, MWIR signal as a function of photodetector–blackbody distance. Right axis, the relationship between the NIR/MWIR ratio and photodetector-blackbody distance. Inset: NIR signal as a function of photodetector–blackbody distance. **b,** NIR/MWIR ratio depends on the blackbody temperature. Red dots show the measured NIR/MWIR ratio in each blackbody temperature.

**8. Crosstalk performance of two-color photodetectors**

Relative spectral crosstalk calculation model is shown in **Fig. S14**. The relative spectral response curves of NIR photodetector and MWIR photodetector are measured respectively, and the two relative spectral response curves are normalized in the same coordinate system. The area *S_A_* of the relative spectral response curves of A band photodetector, the area *S_B_* of the relative spectral curves of B band photodetector, the wavelength axis, and the overlap areas *S^`^_A_* and *S^`^_B_* are calculated respectively. Therefore, the crosstalk formula is:

$C_{MWIR\text{ }to\text{ N}IR}=\frac{S_{B}^{'}}{S_{A}}\times100\%$ (9)

$C_{NIR\text{ }to\text{ }MWIR}=\frac{S_{A}^{'}}{S_{B}}\times100\%$ (10)

We also test the crosstalk and response of light-doped Si two-color photodetectors with different bias voltages under 2 μm light (**Fig. S15**). The results show that different bias voltage in NIR does not affect the working state of MWIR region. Therefore, when the device is suitable for working under NIR reverse bias, combining photoresponse and crosstalk to finally choose the working bias of NIR device as -0.5 V.

Heavy doping Si two-color device, a lot of crosstalk appeared in the test process. The operating conditions of the device under different bias voltages are tested (**Fig. S16**). Due to the high doping concentration of heavily doped Si, the overall energy band change of the device produces a lot of electrical crosstalk itself. The test results show that the doping concentration of Si can affect the electrical crosstalk of the device.


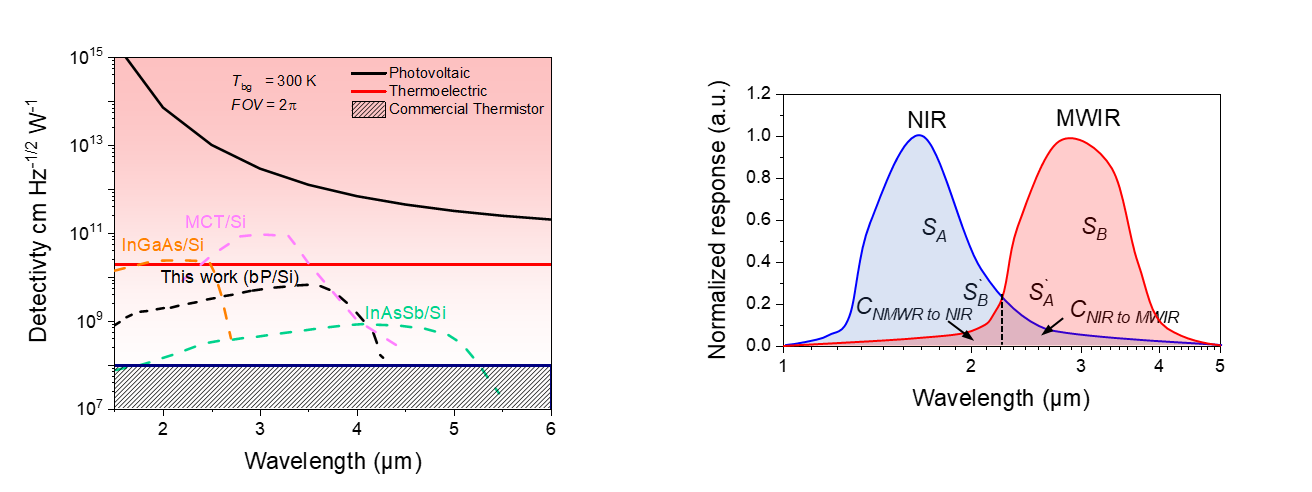


**Fig. S14.** **Schematic diagram of crosstalk in the normalized spectra response of NIR/MWIR photodetectors.**

**Fig. S15. Crosstalk changes of different work bias in light doping silicon.** The red line is MWIR device and the black line is NIR device.

**Fig. S16.** **Crosstalk changes of different work bias in heavily doping silicon.** The red line is MWIR device and the black line is NIR device.

**9. Heat source imaging and temperature measurement.**

We image the heat source and successfully tested the temperature according to the photocurrent information. The two-color device can eliminate the emissivity and other intermediate interference and lock the heat source temperature through the spectrum. The hand-held thermometer can only identify the heat source glass shell temperature about 700 K by the thermistor (**Fig. S17**).

Because the heat source is carbon fiber electric heating wire wrapped in vacuum glass, the contact thermometer cannot be used to measure the temperature. The manufacturer has informed that the temperature of the internal electric heating wire is about 1300 K. The van der Waals two-color photodetector is measured at 1257 K, which is close to the actual temperature.

**Fig. S17. Temperature test results of hand-held thermometer.**
